# COVID-19 and the transition to virtual teaching sessions in an orthopaedic surgery training program: a survey of resident perspectives

**DOI:** 10.1186/s12909-022-03703-1

**Published:** 2022-09-01

**Authors:** Colin Kruse, Kyle Gouveia, Patrick Thornley, James R. Yan, Colm McCarthy, Teresa Chan, Waleed Kishta, Vickas Khanna

**Affiliations:** 1 Michael G. DeGroote School of Medicine, McMaster University, Hamilton, ON Canada; 2 Department of Surgery, Division of Orthopaedic Surgery, McMaster University, ON Hamilton, Canada; 3 Department of Medicine, Division of Emergency Medicine, McMaster University, ON Hamilton, Canada

**Keywords:** Virtual teaching, Online teaching, Orthopaedic surgery, Residents, Surgery, COVID-19

## Abstract

**Background:**

COVID-19 has had a tremendous impact on medical education. Due to concerns of the virus spreading through gatherings of health professionals, in-person conferences and rounds were largely cancelled. The purpose of this study is the evaluate the implementation of an online educational curriculum by a major Canadian orthopaedic surgery residency program in response to COVID-19.

**Methods:**

A survey was distributed to residents of a major Canadian orthopaedic surgery residency program from July 10^th^ to October 24^th^, 2020. The survey aimed to assess residents’ response to this change and to examine the effect that the transition has had on their participation, engagement, and overall educational experience.

**Results:**

Altogether, 25 of 28 (89%) residents responded. Respondents generally felt the quality of education was superior (72%), their level of engagement improved (64%), and they were able to acquire more knowledge (68%) with the virtual format. Furthermore, 88% felt there was a greater diversity of topics, and 96% felt there was an increased variety of presenters. Overall, 76% of respondents felt that virtual seminars better met their personal learning objectives. Advantages reported were increased accessibility, greater convenience, and a wider breadth of teaching faculty. Disadvantages included that the virtual sessions felt less personal and lacked dynamic feedback to the presenter.

**Conclusions:**

Results of this survey reveal generally positive attitudes of orthopaedic surgery residents about the transition to virtual learning in the setting of an ongoing pandemic. This early evaluation and feedback provides valuable guidance on how to grow this novel curriculum and bring the frontier of virtual teaching to orthopaedic education long-term.

**Supplementary Information:**

The online version contains supplementary material available at 10.1186/s12909-022-03703-1.

## Background

The emergence of the novel coronavirus and its associated disease (COVID-19) was declared a pandemic by the World Health Organization (WHO) on March 11^th^, 2020 [1], and has since led to numerous changes to the field of orthopaedic surgery [2–7]. The novel coronavirus has also forced a paradigm shift in the way medical education is delivered [8–11]. Due to concerns of the virus spreading amongst healthcare workers, in-person conferences and teaching rounds were largely converted to online offerings [11], and many residency programs have transitioned to online educational curriculums out of necessity [9, 10]. Specifically, surgical residency programs required drastic changes to find options for procedural training, as well as ways to continue high-quality teaching [12–15].

Recent events related to COVID-19 have caused an immense increase in the utilization of virtual learning. With this recent explosion, the literature has revealed many advantages compared to traditional in-person teaching sessions, including the ability to connect learners from different geographic locations, ease of access to material, and the ability to invite speakers from all over the world [16, 17]. However, the impact of virtual learning on resident education, and the attitudes of learners towards this transition to such a learning model remain unclear.

The purpose of this study is to evaluate the implementation of an online educational curriculum by a major Canadian orthopaedic surgery residency program in response to COVID-19, and to examine how the transition to virtual teaching has impacted residents’ participation, engagement, and overall educational experience.

## Methods

We conducted a survey-based study to evaluate the perceptions of orthopedic trainees about their recent shift to online learning. Importantly, the transition from in-person to the online learning format did not represent a change to the core orthopedic curriculum content of the postgraduate training program surveyed. We received an exemption from our institutional review board to conduct this program evaluation study.

### Survey development

A pilot survey was created by one of the authors (JY) and then distributed to a local expert group consisting of the residency program education director (WK), residency program director (VK), and a local medical education research expert (TC) for feedback regarding survey content, comprehensiveness, and clarity. Emphasis was placed on questions designed to elicit demographic data and to gauge respondents’ familiarity with virtual education prior to its implementation, as well as examine how virtual learning has impacted their participation, engagement, and overall educational experience. A complete version of the final survey can be found in Additional file 1: Appendix 1. The raw data from the survey can be found in Additional file 2: Appendix 2.

### Survey administration

The survey was administered online using SurveyMonkey® (www.surveymonkey.com, Palo Alto, CA). The survey was distributed to all twenty-eight residents of a major Canadian orthopaedic surgery training program six months after the implementation of virtual grand rounds and teaching sessions. Requests for participation were sent via two batch e-mail communications from the selected orthopaedic surgery department communications director. The survey was distributed electronically on July 10, 2020. Responses for the survey were closed on October 24, 2020 and were limited to a single survey response per individual. At the end date of the survey, data was exported to a secure Microsoft Excel spreadsheet (Version 14.7, Microsoft, Redmond, WA, USA) prior to data analysis.

### Statistical analysis

The statistical analysis plan was established *a priori*. All responses to the survey were reported in descriptive statistics, using a proportions or mean ± standard deviation (SD) as appropriate. Likert scale categories were collapsed due to shared directionality as appropriate (e.g. More and Significantly More).

## Results

### Demographics

In total, 25 surveys (response rate of 89%), were completed and analyzed (see Table 1). This included a 100% survey response rate from residents in their first, second and third year of residency. The greatest number of responses were submitted by third year residents (*n* = 11, 100% 44%), followed by first year residents (*n* = 6, 100% 24%) and second year residents (*n* = 4, 100% 16%).

**Table 1:** Resident responses comparing the virtual teaching seminars to the in-person seminars (N, number)

	1 Significantly less N (%)	2 Less N (%)	3 The same N (%)	4 More N (%)	5 Significantly more N (%)
Quality of education	0 (0)	2 (8)	5 (20)	15 (60)	3 (12)
Level of engagement	0 (0)	4 (16)	5 (20)	13 (52)	3 (12)
Knowledge acquisition	0 (0)	2 (8)	6 (24)	12 (48)	5 (20)
Diversity of teaching topics	0 (0)	0 (0)	3 (12)	14 (56)	8 (32)
Variety of presenters	0 (0)	0 (0)	1 (4)	14 (56)	10 (40)
Meeting personal learning objectives	0 (0)	3 (12)	3 (12)	16 (64)	3 (12)
Faculty engagement	0 (0)	5 (20)	3 (12)	12 (48)	5 (20)
Faculty attendance	0 (0)	3 (12)	2 (8)	16 (64)	4 (16)

Prior to the implementation of this online curriculum, five respondents (20%) had never participated in virtual learning before, while another fourteen respondents (56%) had not done so recently. Prior to this experience, sixteen respondents (64%) felt they were totally unfamiliar or somewhat unfamiliar with virtual teaching.

### Current participation

All respondents were taking part in daily online morning teaching rounds at the time of survey distribution. The majority of respondents were also taking part in grand rounds (*n* = 23, 92%), journal clubs (*n* = 20, 80%), and third-party educational webinars (*n* = 18, 72%) through various e-platforms on a weekly basis. The most common platform used for virtual education was Zoom (*n* = 25, 100%), followed by Webex (*n* = 12, 48%) and Microsoft Teams (*n* = 11, 44%).

### Comparison to in-person seminars

Compared to in-person sessions, 18 residents (72%) found the quality of education via online teaching rounds to be superior, with 64% feeling that their level of engagement improved. In comparison to in-person seminars, 68% of respondents felt that they were able to acquire more knowledge during virtual seminars, 88% felt that there was a greater diversity of teaching topics, and 96% appreciated an increased variety of presenters. Overall, 76% of respondents felt that the virtual seminars better met their personal learning objectives. When asked to choose which format they ultimately prefer, 13 respondents (52%) chose virtual teaching.

### Verbatim comments

Respondents were asked to provide commentary regarding advantages and disadvantages of the new virtual seminars for learners, which are displayed in Table 2.

**Table 2:** Descriptive comments from the residents on the online teaching rounds

Category	Comment
Advantages of Virtual Seminars	“I find myself much more focused when watching the lectures through my screen. It feels like a personalized teaching session as opposed to an auditorium, which I find distracting. Also, the convenience of meeting over zoom makes attendance much easier as you don't have to commute.”
	“Eliminating the need to travel on site early in the morning, thus allowing more rest and more focus when learning.”
	“The ability to record and less time travelling in a car. Presenters tend to prepare videos for their presentations.”
	“It is much easier to have high profile speakers from around the world, and they are more convenient.”
Disadvantages of Virtual Seminars	“There is less dynamic feedback from the audience.”
	“It is easier to tune out and hide.”
	“There is a loss of physical interactions with fellow classmates and residents. The social aspect is lacking.”
	“There is more screen time and a little less interactivity from the audience.”
Suggestions for improvement	“Include more polls and open talk times to improve engagement.”
	“Develop methods for the presenter to obtain feedback from the audience.”
	“Encourage more back and forth between the presenter and the audience right from the beginning of the session.”

## Discussion

The major finding of this cross-sectional survey is that resident feedback on the new curriculum was overwhelmingly positive. Compared to the in-person sessions, the vast majority of resident respondents surveyed felt like the quality of education that they received through online morning teaching rounds was superior.

Notably, most residents felt that they were more engaged with the presented content in comparison to in-person seminars. This is consistent with medical education literature pertaining to other specialties, with a blended learning model for radiology demonstrating that medical students had higher levels of performance, satisfaction, and engagement [18]. Increased engagement may be attributable in part to improved accessibility, as residents presently surveyed acknowledged that teaching rounds were easier to attend because the need for travel was effectively eliminated. Furthermore, residents are able to re-watch recorded virtual teaching rounds. There is an established body of evidence that supports the idea that the key to adult human learning is spaced repetition [19, 20] and these effects have also been demonstrated in orthopaedic surgery residents [21]. In addition, residents found the virtual presentations to be better prepared and more personalized, and also noted the benefits of having high profile speakers from around the world join with ease (Table 2).

Given that the novel coronavirus pandemic is a recent development and the transition to virtual learning for many surgical programs has occurred, there is limited published literature on resident perspectives on this new format of learning. In a recent study, Figueroa *et al.* found that online webinars were very highly rated by orthopaedic residents and when asked if they would continue participating in these online webinars after the pandemic had stopped, 82% answered that they would [11]. Similarly, in neurosurgery, Al-Ahmari *et al.* found that 75% of the residents surveyed felt that online lectures were more useful than the traditional in-person lectures [10]. These findings are not limited to surgical residents, as Rana *et al.* found that 82% of senior residents in surgical and medical specialties reported that virtual lectures were more effective than in-person lectures [22]. Interestingly, the majority of our respondents felt a level of unfamiliarity with virtual platforms at the time of transition. It is possible that there are even greater benefits to be gained after further experience with these platforms. Our present study provides more early support to the notion that surgical residents view the new virtual seminars positively, as residents felt that they were able to gain more knowledge and better achieve their personal learning objectives.

Although the feedback was primarily positive, one drawback identified was decreased presenter and audience interaction, which afforded the presenter less dynamic feedback. Furthermore, some residents felt the online sessions were less personal and lacked the enjoyable social aspects of in-person rounds. Despite most residents (72%) saying they felt the education received was superior, only 52% of residents said they would choose them over the in-person rounds. It is possible that some combination of the two formats of teaching may be ideal in the future. This information is especially becoming important as the pandemic continues to evolve both in North America and beyond. As many jurisdictions see less public health measures restricting gatherings, decisions will have to be made regarding the benefits of virtual presentations even when not necessitated by those same measures. Our study supports that though residents appreciate the social aspect of gathering in person, the benefits of virtual education sessions make the case for them to continue at least in some form going forward.

A main limitation in this study is the single orthopaedic surgery department trainee assessment of a relatively small number of respondents despite a high response rate. It is unclear whether the results would be replicated in other programs, or even other surgical specialties. A further limitation of the study is failure to capture residents who participated in the virtual learning curriculum from March 2020 to their graduation in June 2020, which likely accounts for a relatively decreased proportion of senior resident respondents. Furthermore, the first-year residents who participated in this study were not able to experience a full year of the curriculum prior to the transition to virtual learning so these results may need to be interpreted with caution. However, these residents would have completed over eight months of training prior to this transition, which should have enabled them to thoroughly compare the two methods. Future research could expand to include the perspective of orthopaedic surgery residents throughout Canada, and with an increased number of respondents it may be possible to analyze differences between subgroups, such as level of postgraduate training. Additionally, with our institutional understanding of high levels of satisfaction with virtual teaching formats, longitudinal assessment and validated examination metrics will be able to be assessed and compared to previous in-person only taught cohorts for the impact of such changes on residency education effectiveness.

## Conclusion

Overall, the results of this cross-sectional survey demonstrate that residents felt the implementation of online teaching rounds resulted in higher quality, more engaging, and more diverse teaching in comparison to in-person counterparts in a large Canadian, academic orthopaedic surgery training program. These findings provide support for the use of online teaching rounds moving forward in orthopaedic surgery residency programs, even as constantly evolving public health measures may begin to play less of a role in necessitating these sessions be done virtually.

## Supplementary Information

**Additional file 1.**

**Additional file 2.**

## Data Availability

All data generated or analysed during this study are included in this published article (and its supplementary information files).

## Abbreviations

COVID-19: Novel coronavirus
WHO: World Health Organization
SD: Standard Deviation
