# Supplementary material for: COVID-19 and the transition to virtual teaching sessions in an orthopaedic surgery training program: a survey of resident perspectives
Source: BMC Med Educ. 2022 Sep 1;22:655. doi: 10.1186/s12909-022-03703-1 (PMC9434512; doi:10.1186/s12909-022-03703-1)
Supplement: Supplementary file 1 — Additional file 1. [file 12909_2022_3703_MOESM1_ESM.pdf]

MacOrtho Evaluation Survey for Digital Teaching Technology

* Required

1. What role best describes you *

Mark only one oval.

- Trainee
- Faculty

2. If selected Trainee above, what is your level of training

Mark only one oval.

- PGY1
- PGY2
- PGY3
- PGY4
- PGY5
- Fellow

3. If selected Faculty above, how many years of independent practice?

Mark only one oval.

- < 5 yrs
- 5 - 10 years
- 10 - 20 years
- > 20 years

4. Which teaching site(s) are you working at (trainees, which teaching site are you currently assigned to) since March, 2020? Select all that apply. *

Check all that apply.

- [ ] St. Joseph's Healthcare
- [ ] Hamilton General Hospital
- [ ] Juravinski Hospital & Cancer Centre
- [ ] McMaster Children's Hospital

5. Having you been taking part in digital teaching seminars since the cancellation of in-person teaching rounds (March, 2020) *

Mark only one oval.

- Yes
- No

6. If yes, which types of seminars have you been attending (select all that apply from below)

Check all that apply.

- Morning teaching rounds
- Grand Rounds
- 3rd party educational webinars
- Journal clubs
- Academic town halls/program meetings

Other: ________________

7. On average, how many hours a week have you been participating in digital teaching seminars *

Mark only one oval.

- < 1 hr
- 2
- 3
- 4
- 5 +

8. Have you participated in digital teaching before? *

Mark only one oval.

- Yes
- Not recently
- Never

9. 9. Prior to this experience, on a scale of 1 to 5, how familiar were you with digital conferences/seminars? *

Mark only one oval.

- 5- Extremely familiar, I would consider myself an expert
- 4- Familiar, I regularly use it
- 3 - Moderately familiar, I have used it before
- 2 - Somewhat unfamiliar, only used it once or twice
- 1 - Totally unfamiliar, never used it before

10. 10. Since starting to use it, on a scale of 1 to 5, how familiar are you with digital conferences/seminars? *

Mark only one oval.

- 5 - Extremely familiar, I would consider myself an expert
- 4 - Somewhat familiar, I am comfortable using it
- 3 - Moderately familiar, I feel like I understand it
- 2 - Somewhat unfamiliar, but getting the hang of it
- 1 - Totally unfamiliar, still very confused about this

11. What platforms have you participated in with digital education? Select all that apply *

Check all that apply.

- [ ] Basecamp
- [ ] Facebook Messenger
- [ ] Google Hangouts/Meet
- [ ] Microsoft Teams
- [ ] WebEx
- [ ] WhatsApp
- [ ] Slack
- [ ] Skype
- [ ] Zoom

Other: [ ] ______________________

12. 13. What format of teaching have you experienced in the digital conferences? Select all that apply *

Check all that apply.

- [ ] Didactic presentation alone
- [ ] Didactic presentation with some method of interaction (online quiz, clicker)
- [ ] Case based discussion directed to a single learner
- [ ] Case based discussion directed to an entire group
- [ ] Expert debate

Other: [ ] ______________________

13. What has been your major role in these digital teaching seminars? *

Mark only one oval.

- Learner *Skip to question 14*
- Presenter/Facilitator *Skip to question 32*

MacOrtho Familiarity Survey to Online Teaching Technology - Learner

14. In comparison to in-person seminars, what is the quality of education you have received in digital seminars? *

Mark only one oval.

- 5 - Far superior
- 4 - Superior
- 3 - The same
- 2 - Inferior
- 1 - Far inferior

15. In comparison to in-person seminars, how has your level of engagement changed with digital seminars? *

Mark only one oval.

- 5 - Far better, much more comfortable with participating
- 4 - Better, easier to follow the material
- 3 - The same
- 2 - Worse, difficult to follow compared to in-person sessions
- 1 - Far worse, cannot pay attention at all

16. In comparison to in person seminars, how do you feel faculty engagement has changed since using digital meetings: *

Mark only one oval.

- Significantly more
- More
- Same
- Less
- Significantly less

17. In comparison to in person seminars, do you feel that faculty have had more or less attendance to teaching/rounds: *

Mark only one oval.

- Significantly More
- More
- Same
- Less
- Significantly Less

18. In comparison to before in-person teaching rounds were cancelled, how many sessions are you getting in a digital format? *

Mark only one oval.

- More
- The same amount
- Less

19. In comparison to in-person seminars, how much knowledge are you able to acquire during digital seminars? *

Mark only one oval.

- Much More
- More
- No change
- Less
- None

20. Prior to this experience, how comfortable were you with reaching out to ask questions? *

Mark only one oval.

- Very comfortable, able to participate at any time
- Somewhat comfortable, able to participate when needed
- Neither comfortable or uncomfortable
- Uncomfortable, minimal participation
- Totally uncomfortable, zero participation

21. After this experience, on a scale of 1 to 5, how comfortable are you with reaching out to ask questions NOW? *

Mark only one oval.

- Very comfortable, able to participate at any time
- Somewhat comfortable, able to participate when needed
- Neither comfortable or uncomfortable
- Uncomfortable, minimal participation
- Totally uncomfortable, zero participation

22. In comparison to in-person seminars, do you find there is more diversity of teaching topics with the digital teaching seminars: *

Mark only one oval.

- 5 - Significantly More
- 4 - More
- 3 - The same
- 2 - Less
- 1 - Significantly Less

23. Do you find that there is more participation by faculty/staff members in digital teaching seminars? *

Mark only one oval.

- Yes
- No
- Unsure

24. In comparison to in-person seminars, what is your opinion on the variety of presenters among digital teaching seminars (different institutions, international) *

Mark only one oval.

- 5 - Significantly More
- 4 - More
- 3 - The same
- 2 - Less
- 1 - Significantly Less

25. In comparison to in-person seminars, how do you find the overall quality of the digital teaching seminars *

Mark only one oval.

- 5 - Significantly More
- 4 - More
- 3 - The same
- 2 - Less
- 1 - Significantly Less

26. In comparison to in-person seminars, how do you feel the digital seminars meet your personal learning objectives *

Mark only one oval.

- 5 - Significantly More
- 4 - More
- 3 - The same
- 2 - Less
- 1 - Significantly Less

27. What have been advantages of digital seminars to deliver educational rounds for learners?

28. What are disadvantages of digital seminars to deliver educational rounds for learners?

29. What have been advantages of in-person sessions to deliver educational rounds for learners?

30. What are disadvantages of in-person sessions to deliver educational rounds for learners?

31. What is your overall experience with digital seminars in comparison to your experience with in-person seminars *

Mark only one oval.

	1	2	3	4	5	
Hate it, never want to do it again						Love it, would never want to any other type of learning again

Skip to question 44

MacOrtho Familiarity Survey to Online Teaching Technology - Presenter

32. In comparison to in-person seminars, what has been your experience in delivering digital rounds? *

Mark only one oval.

- 5 - Much easier/less work
- 4 - Easier/less work
- 3 - The same
- 2 - Some more work/difficult
- 1 - Much more work/more difficult

33. In comparison to in-person seminars, what do you perceive the level of resident engagement in digital seminars *

Mark only one oval.

- 5 - Much more participation
- 4 - More participation/engagement
- 3 - The same amount
- 2 - Very little, had to prompt at lot
- 1 - Far less, virtually no participation

34. In comparison to in-person seminars, how well were you able to assess learner's level of knowledge or understanding during digital seminars *

Mark only one oval.

- 5 - perfectly able to ascertain
- 4 - easier to assess
- 3 - no difference
- 2 - difficult to assess
- 1 - completely unable to assess

35. How well do digital seminars allow you to communicate directly with residents compared to in-person teaching? *

Mark only one oval.

- 5 - Much better
- 4 - Somewhat better
- 3 - The same
- 2 - Somewhat worse
- 1 - Much worse

36. Since starting digital meetings I feel... *

Mark only one oval.

- 5 - Significantly more engaged in resident teaching
- 4 - More engaged in resident teaching
- 3 - Same level of engagement
- 2 - Less engaged in resident teaching
- 1 - Significantly less engaged in resident teaching

37. Do you feel the digital meetings compared to in person teaching allow you to reach your teaching objectives *

Mark only one oval.

- 5 - Significantly more
- 4 - More
- 3 - Same
- 2 - Less
- 1 - Significantly less

38. Have you noticed more participation/attendance from other faculty/staff members in digital teaching seminars? *

Mark only one oval.

- Yes
- No
- Unsure

39. What have been advantages of digital seminars to deliver educational rounds for teachers?

40. What are disadvantages of digital seminars to deliver educational rounds for teachers?

41. What have been advantages of in-person sessions to deliver educational rounds for teachers?

42. What are disadvantages of in-person sessions to deliver educational rounds for teachers?

43. Overall did you enjoy teaching using a digital seminar experience? *

Mark only one oval.

- 7 - Greatly enjoyed it, this should be the standard moving forward
- 6 - Enjoyed it, generally worked well
- 5 - Mildly enjoyed it, still room for improvement
- 4 - Neither liked nor disliked it
- 3 - Mildly disliked it, but fixable
- 2 - Disliked it, would strongly consider not doing this again
- 1 - Totally hated it, would never do this again.

Skip to question 44

MacOrtho Familiarity Survey to Online Teaching Technology pg 2

44. Since starting digital meetings I feel that my wellness has *

Mark only one oval.

- Significantly improved
- Improved a little bit
- Remained the same
- Worsened a little bit
- Significantly worsened

45. What can be done to improve digital teaching seminars?

46. What can be done to improve in person presentations

47. If you had to pick one, which type of rounds would you prefer? *

Mark only one oval.

- ( ) Digital teaching
- ( ) In-person

48. Follow up from the previous question, why?

This content is neither created nor endorsed by Google.

Forms
